# Mosquito Species Diversity and Circulation of Mosquito-Borne Viruses in Selected Provinces of Central Vietnam

**DOI:** 10.3390/v17070905

**Published:** 2025-06-26

**Authors:** Margarita R. Popova, Alena A. Sharova, Anna S. Gladkikh, Tatiana V. Arbuzova, Ekaterina O. Klyuchnikova, Valeriya A. Sbarzaglia, Nadezhda A. Tsyganova, Dmitry D. Naydenov, Anastasia S. Gritseva, Edward S. Ramsay, Regina R. Baimova, Islam A. Karmokov, Ekaterina. G. Riabiko, Nikolai K. Tokarevich, Nguyen T. Dong, Bui T. Phu, Vu T. Phan, Do T. Hung, Trinh C. Thuc, Vladimir G. Dedkov

**Affiliations:** 1 Saint Petersburg Pasteur Institute, 197101 Saint Petersburg, Russia; alenasharova21@gmail.com (A.A.S.); angladkikh@gmail.com (A.S.G.); arbuzowa95@yandex.ru (T.V.A.); ekaterina.ibg@gmail.com (E.O.K.); sbarzaglia.valeriya@gmail.com (V.A.S.); nadya-ts@mail.ru (N.A.T.); dmitnayd@gmail.com (D.D.N.); gritseva42@gmail.com (A.S.G.); baimova@pasteurorg.ru (R.R.B.); karmokov@pasteurorg.ru (I.A.K.); riabiko@pasteurorg.ru (E.G.R.); zoonoses@mail.ru (N.K.T.); vgdedkov@pasteurorg.ru (V.G.D.); 2 Pasteur Institute in Nha Trang, Nha Trang 650000, Vietnam; dongpasteur@gmail.com (N.T.D.); phu79pasteur@gmail.com (B.T.P.); phanvutienpas@gmail.com (V.T.P.); hungdt02@yahoo.com (D.T.H.); thucpasteur83@gmail.com (T.C.T.); 3 E. I. Martsinovsky Institute of Medical Parasitology, Tropical, and Vector Borne Diseases, Sechenov First Moscow State Medical University, 119992 Moscow, Russia

**Keywords:** arboviruses, dengue virus, mosquitoes species diversity, *Aedes* spp.

## Abstract

Arboviruses, including but not limited to dengue virus (DENV), Zika virus (ZIKV), and chikungunya virus (CHIKV), pose a significant global threat to human health. The transmission of DENV, ZIKV, and CHIKV is facilitated by mosquitoes belonging to the genus *Aedes*, which are prevalent in both urban and rural regions of Vietnam. In 2023 an investigation into the population of mosquitoes was conducted in a number of provinces located within the central region of Vietnam. A total of 12,546 mosquitoes were collected during the study. The mosquitoes collected comprised the genera *Culex* spp., *Aedes* spp., *Anopheles* spp., and *Armigeres* spp. The *Aedes* spp. mosquitoes were predominant, being collected in 908 pools. These were then examined by RT-qPCR for the detection of DENV, ZIKV, and CHIKV. DENV viral RNA was detected in 92 mosquito pools, ZIKV was detected in 1 mosquito pool, and CHIKV was not detected. The typing of samples containing DENV RNA was carried out. It is evident from the results of the typing process that three distinct types of DENV have been identified. The three main dengue virus types are DENV-1, DENV-2, and DENV-4.

## 1. Introduction

Arboviruses, short for arthropod-borne viruses, are a diverse group of pathogens transmitted by arthropods such as mosquitoes, ticks, and sandflies. These viruses are a growing global health concern, especially in tropical and subtropical regions where the climate and environment are conducive to the proliferation of their vectors. For the moment, over 500 arboviruses have been identified, with more than 130 known to infect humans [1]. These viruses belong to several families, including *Flaviviridae*, *Togaviridae*, *Bunyaviridae*, and *Reoviridae*. Diseases caused by arboviruses such as dengue fever, Zika fever, and chikungunya fever affect millions of people each year, placing a serious health and economic burden on affected regions. The ability of arboviruses to spread across continents and affect diverse populations makes them a persistent and escalating public health challenge [2].

According to the World Health Organization (WHO), dengue fever stands as the most prevalent arboviral disease worldwide, posing a significant threat to half of the global population. Dengue fever alone is estimated to cause between 100 and 400 million infections each year, with approximately 100 million resulting in symptomatic illness [3,4].

Dengue virus (DENV) is transmitted by *Aedes aegypti* (*A. aegypti*) and *Aedes albopictus* (*A. albopictus*) mosquitoes, which are found in more than 100 countries across tropical and subtropical regions [5]. Southeast Asia, in particular, bears a significant portion of this burden, with Vietnam emerging as one of the countries most affected by frequent dengue outbreaks. According to the WHO, Vietnam reported over 149,557 dengue infections and 36 deaths in 2023. This underscores the severity of the situation, particularly in the southern and central regions where climatic conditions are most favorable for mosquito proliferation [4]. Dengue fever outbreaks in Vietnam follow a seasonal pattern, with peak transmission occurring during the rainy season from May to October. This period is characterized by an increase in mosquito populations, especially *A. aegypti*, the primary vector for DENV [6].

The increasing risk of dengue fever epidemic spread is associated with several factors. These include the following: changing distribution of the vectors (chiefly *A. aegypti* and *A. albopictus* mosquitoes); climate change leading to certain conditions (higher temperatures, rainfall, and humidity); and fragile health systems in the midst of the COVID-19 pandemic. Other factors also play a role, such as political or financial instabilities in certain countries facing complex humanitarian crises or high population movement. Urbanization is associated with DENV transmission through multiple social and environmental factors. These include population density, human mobility, access to reliable water sources, and water storage practices. Vectors may adapt to new environments and climates, and the interactions between the virus, the host, and the environment are dynamic. Consequently, disease risks may change and shift in association with the aforementioned factors: climate change in certain tropical and subtropical areas; increased urbanization; and population movement [7,8].

DENV includes four antigenically distinct types (DENV-1, DENV-2, DENV-3, and DENV-4). All four dengue types circulate in Vietnam, with shifts in their prevalence over time. Historically, DENV-1 and DENV-2 have been the dominant types. Recent studies, however, have indicated an increase in DENV-3 and DENV-4 cases, particularly in the southern and central regions of Vietnam [9]. The challenge of managing dengue fever is compounded by circulation of all four DENV types, which complicates both clinical management and preventive measures. The risk of severe dengue fever, including dengue hemorrhagic fever (DHF) and dengue shock syndrome (DSS), is heightened by the phenomenon of antibody-dependent enhancement (ADE), which occurs when individuals are reinfected with a different DENV type following a primary infection [10]. The presence of multiple DENV types circulating simultaneously increases the likelihood of secondary infections. This, in turn, raises the risk of more severe outbreaks. These shifts in DENV type dominance are also observed in other dengue-endemic regions, where changes in serotype patterns complicate outbreak prediction and control efforts [11].

Zika virus (ZIKV, family *Flaviviridae*) can be transmitted to humans through *Aedes* mosquitoes as a vector [12,13,14,15]. The first human case of ZIKV infection was reported in 1954 in Nigeria, and sporadic cases have been noted in Asia [16]. It has been widely reported that approximately 80% of people with ZIKV infection are asymptomatic. Although the disease is self-limiting, cases of neurological manifestations have been described. Between 2015 and 2016, ZIKV was a global health concern following large outbreaks in the Americas [15,16,17].

The chikungunya virus (CHIKV), an alphavirus first isolated in Tanzania in 1953, has been detected since the 1960s in Asia and was reported in Vietnam in 1967 [18,19]. In 2004, a new variant of CHIKV emerged in East Africa and rapidly spread throughout the Indian Ocean region, India, and Thailand, causing significant outbreaks [19,20,21,22]. The early symptoms of dengue fever, Zika fever, and chikungunya fever are highly similar. This can lead to confusion and underestimation of the burden of chikungunya fever [23]. Furthermore, the same *Aedes* mosquito species are vectors for all three viruses, and such species are widely prevalent in the region.

The present study was prompted by the significant disease burden caused by arboviruses carried by *Aedes* mosquitoes. It aims to further our understanding of vector population dynamics in specific provinces of Vietnam. The findings of this study have expanded the extant body of knowledge on the diversity and percentage of different mosquito species. Furthermore, this study enabled estimation of the infection prevalence and distribution of different DENV types among *Aedes* mosquitoes in certain provinces of Vietnam.

## 2. Materials and Methods

### 2.1. Field Sampling and Species Determination

**Figure 1:**
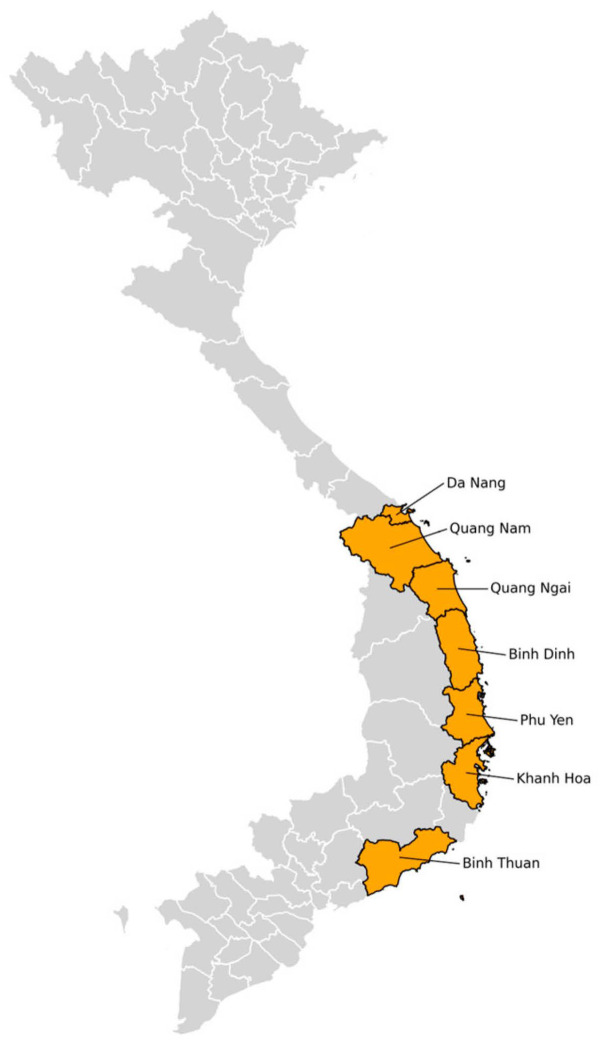
Vietnamese provinces wherein samples were collected. GADM v4.0, GeoPandas v1.0.1, Matplotlib v3.7.0 [26].

Samples of adult mosquitoes were collected in three phases: January and February 2023; May and June 2023; and October and November 2023. Mosquitoes were collected in seven provinces: Da Nang, Quang Nam, Quang Ngai, Binh Dinh, Phu Yen, Khanh Hoa, and Binh Thuan (Figure 1). Adult mosquitoes were collected from households and from water containers inside and outside the house. Mosquitoes were collected with a CDC Backpack Aspirator [24]. Adult mosquitoes were then transported to the laboratory at the Pasteur Institute in Nha Trang. Mosquitoes were classified according to the “Illustrated Key to the Mosquitoes of Vietnam” by Chester J. Stojanovic and Harold George Scott [25].

### 2.2. Preparation of Mosquito Samples and RNA Extraction

Female mosquitoes of the genus *Aedes* were screened for further PCR studies. The *Aedes* female mosquitoes were then pooled, with each pool containing 2–10 individuals based on species, collection location, and collection date. A total of 882 pools of *A. aegypti* (representing 2957 mosquitoes) and 26 pools of *A*. *albopictus* (representing 65 mosquitoes) mosquitoes were sampled in the laboratory for the purpose of DENV, CHIKV, and ZIKV testing.

Collected samples were washed using 70% ethanol to prevent surface contamination and rinsed twice in a sterile phosphate-buffered saline (PBS) solution (Sigma-Aldrich, Petaling Jaya, Selangor, Malaysia). Each mosquito pool was homogenized in 2 mL microcentrifuge tubes (Eppendorf, Hamburg, Germany) with stainless steel beads (QIAGEN, Hilden, Germany) and 500 microliters (μL) of 1 × PBS (Sigma-Aldrich, Selangor, Malaysia). Homogenization was performed using a TissueLyser LT homogenizer (QIAGEN, Hilden, Germany). The supernatant (450 μL) was transferred into 2 mL microcentrifuge tubes for further RNA isolation and storage.

RNA samples were obtained by extraction and purification using the AmpliSens^®^ RIBO-Prep^®^ (AmpliSens, Moscow, Russia) reagent kit for total RNA/DNA isolation according to manufacturer recommendations. Samples were eluted with 60 µL of RNA-Buffer^®^ (AmpliSens, Moscow, Russia) and stored at −70 °C until further analysis.

### 2.3. Molecular Studies Based on Real-Time PCR

Nucleic acids from mosquito samples were analyzed for the presence of DENV and ZIKV using the AmpliSens^®^ Dengue virus-FL and Zika virus-FL real-time PCR (AmpliSens, Moscow, Russia) kits in accordance with manufacturer instructions. DENV typing was conducted using RT-qPCR, with primers and amplification conditions previously outlined by Santiago et al. [27]. For DENV type identification, 92 RNA-positive sample pools were analyzed. Nucleic acids isolated from the mosquito samples were also analyzed for the presence of the CHIKV virus. Analysis was conducted using RT-qPCR with specific oligonucleotides and amplification conditions described in a previous study by Waggoner et al. [28]. PCR was performed using a CFX96 C1000 TouchTM (Bio-Rad, Hercules, CA, USA) instrument (Bio-Rad, USA). In order to exclude non-specific reactions, negative controls were utilized. Specifically, a negative extraction control and a negative PCR control were used.

### 2.4. Spatial Data and Visualization

Administrative boundaries (Admin 1) for Vietnam were obtained from the GADM database v4.0 [26]. These boundaries were stored in shapefiles, a common format for representing geographic information. To handle and process these shapefiles, we used GeoPandas v1.0.1 [29], which is a Python 3.13.1 library designed to work with spatial data. The library simplifies reading, manipulating, and analyzing geospatial data. Processed data was then plotted using Matplotlib v3.7.0 [30], a widely used Python plotting library that creates static, interactive, and animated visualizations. The provinces of interest were sampled and highlighted on the map, with labels placed based on the centroid coordinates of each province.

### 2.5. Data Aggregation and Plotting

**Figure 2:**
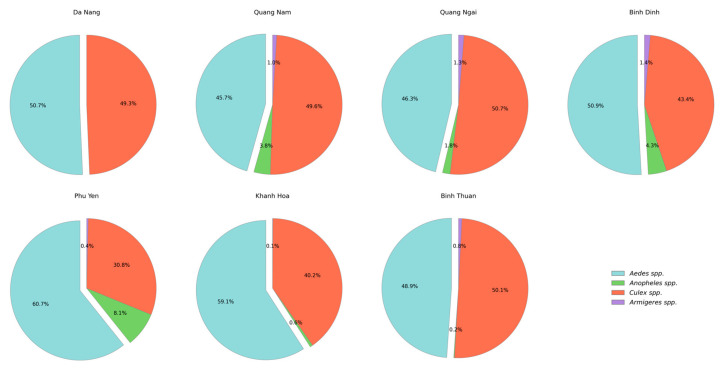
Mosquito species diversity as percentage.

Mosquito species counts were aggregated using Pandas v2.0.3 [31], a Python library for data manipulation. Pandas allowed us to group, sum, and analyze data effectively. To visualize the species diversity (Figure 2), Matplotlib was used to generate pie charts, providing a clear overview of species distribution. The sex distributions of *A. aegypti* and *A. albopictus* were plotted as grouped bar charts (Figure S1), making it easy to compare the numbers of male and female mosquitoes within each species across different samples.

**Figure 3:**
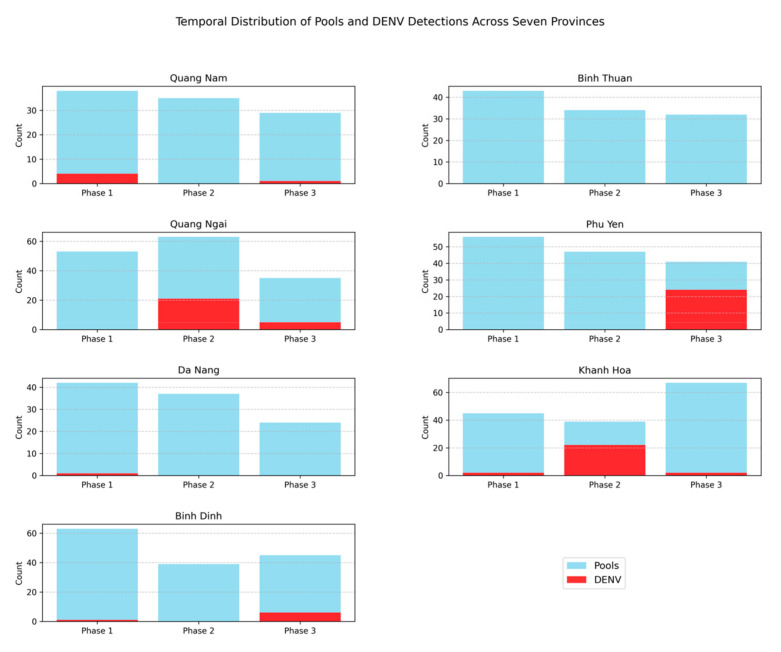
Distribution of DENV-positive mosquito pools among all collected pools by province (2023). Bar plots of total mosquito pools (blue) and DENV-positive pools (red) across three sampling phases in each province (pandas v2.0.3, Matplotlib v3.7.0).

**Figure 4:**
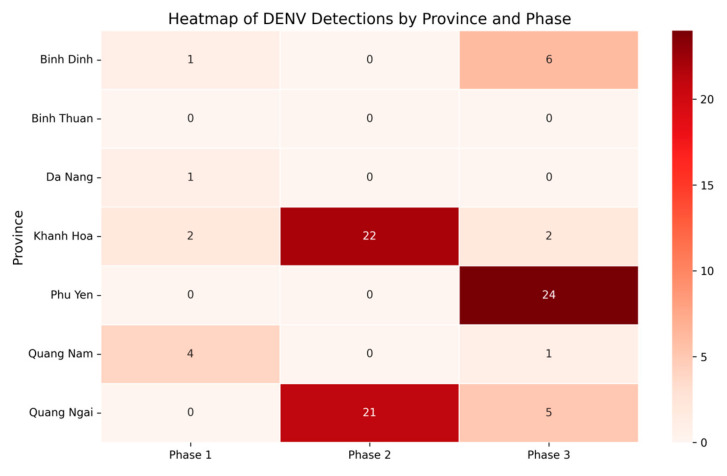
Heatmap of DENV detections by province (pandas v2.0.3, Seaborn v0.12.2).

**Figure 5:**
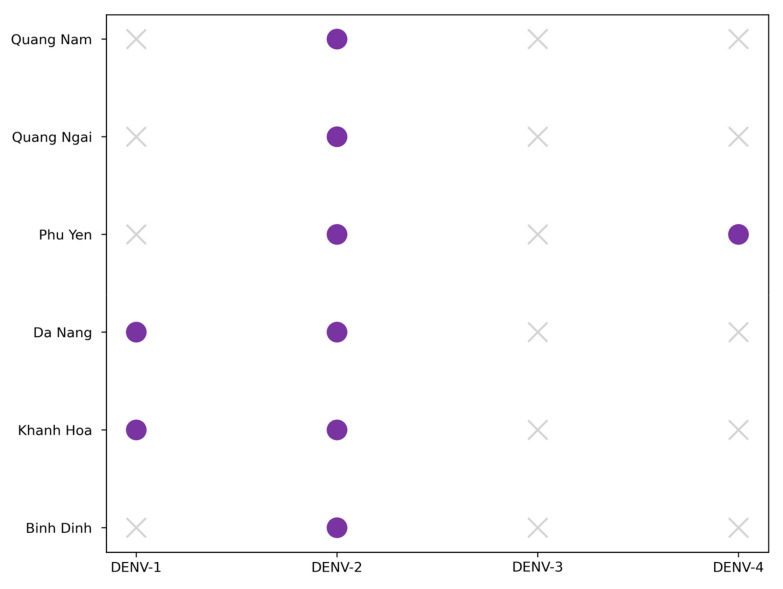
DENV types by province. Presence of DENV serotypes (DENV-1 to DENV-4) across sampled provinces (pandas v2.0.3, Matplotlib v3.7.0).

Additionally, total and DENV-positive pool counts across provinces and phases were visualized using bar plots (Figure 3). For analyzing DENV detection, we summarized the data in pivot tables (a Pandas function that restructures data for easier analysis) and visualized the results using heatmaps (Figure 4 and Figure S2) with Seaborn v0.12.2 [32]. The latter is a statistical data visualization library built on Matplotlib that makes it easier to generate complex plots. Finally, DENV serotype detection was visualized with color-coded scatter plots (Figure 5), again using Matplotlib to display the relationships between serotype occurrence and other variables.

## 3. Results

### 3.1. Species Composition Collected in the Central Region, 2023

In six provinces (Quang Nam, Quang Ngai, Binh Dinh, Phu Yen, Quang Nam, and Khanh Hoa), mosquito specimens were collected representing four species: *Culex*; *Anopheles*; *Aedes*; and *Armigeres*. In Da Nang, only two mosquito species were collected, *Aedes* and *Culex*, which were the most common species in all seven sampling areas. The numbers of mosquitoes collected over three study phases in selected provinces (central region) are presented in Supplementary Materials Table S1. The total number of *Aedes* mosquitoes collected was 6430. Among them, 6323 were identified as *A. aegypti* and 107 as *A. albopictus*. Other genera included in the study were *Culex* spp. (5640 individuals), *Anopheles* spp. (374 individuals), and *Armigeres* spp. (102 individuals). The mosquito species composition profiles identified are presented in Figure 2, as well as Supplementary Materials Table S1.

A total of 3408 male *Aedes* mosquitoes were collected. The number of male *A. aegypti* (3366 total) in the provinces was very high, ranging from 303 to 674 individuals at sampling sites. *A. albopictus* males were identified in two provinces, Binh Dinh (40 individuals total) and Quang Nam (2 individuals total), across all three collection phases (Supplementary Materials—Figures S1 and S2).

A total of 3022 female *Aedes* mosquitoes were collected. The numbers of female *A. aegypti* (2957 total) collected in various localities were different. Da Nang featured the least, while the highest number was collected in Quang Ngai (292 and 519 individuals, respectively). Furthermore, the number of *A. albopictus* females (65 total) was found to be the highest in Binh Dinh and the lowest in Da Nang (25 and 2 total individuals, respectively). Further information on pools and their species compositions are given in Supplementary Materials Figures S1 and S2.

### 3.2. Prevalence of DENV, ZIKV, and CHIKV in Aedes Mosquitoes

A total of 908 *Aedes* mosquito pools were analyzed. DENV viral RNA was detected in 92 of these, representing 10.1% of all samples studied. CHIKV was not detected. ZIKV was detected in one sample from Quang Nam Province. In this study, 340 *Aedes* mosquito pools (representing 1031 mosquitoes) were examined in the first phase of mosquito collection (January–February, 2023). The percentage of positive pools was 2.4%. The highest infection prevalence was observed in Quang Nam Province (10.5%). In Khanh Hoa Province, it was 4.4%. In Da Nang Province, it was 2.4%. The lowest infection prevalence was observed in Binh Dinh Province. DENV was not detected in Binh Thuan, Quang Ngai, or Phu Yen provinces.

In the second phase of mosquito collection (May–June, 2023), 295 *Aedes* mosquito pools (representing 1090 mosquitoes) were examined. The percentage of positive pools was 14.6%. DENV was detected in two provinces: Khanh Hoa and Quang Ngai. Their DENV prevalence values were 56.4% and 33.3%, respectively.

In the third phase of mosquito collection (October–November, 2023), 273 *Aedes* mosquito pools (representing 901 mosquitoes) were examined. The percentage of positive pools was 14.7%. In the third phase of mosquito collection, dengue virus was detected in all provinces except Binh Thuan. The highest prevalence values were observed in Phu Yen (58.5%), Quang Ngai (14.3%), and Binh Dinh provinces (13.3%) (Figure 3 and Figure 4).

During the study, no DENV, ZIKV, or CHIKV viral RNA was detected in *A. albopictus* mosquito pools. There were also no cases of co-infection with viruses such as ZIKV and DENV in the pools.

### 3.3. Dengue Typing

DENV typing was performed on 92 samples containing DENV RNA. The specific dengue type was determined for 57 samples. The predominant types were DENV-2 (71.9%) and DENV-1 (26.3%). DENV-4 was detected in one sample, representing 1.8% of the total number of typed samples. In three provinces (Binh Dinh, Quang Nam, and Quang Ngai), DENV-2 alone was detected. DENV-1 and DENV-2 were detected in Da Nang and Khanh Hoa provinces. DENV-2 and DENV-4 were detected in Phu Yen Province. Figure 5 summarizes detection data by province. Typing failed in 35 cases, likely due to insufficient viral RNA as evidenced by elevated Ct values (>30) in RT-qPCR assays.

The results indicate that there was no simultaneous infection with several DENV types in the pools.

## 4. Discussion

Vietnam is one of the tropical Asian countries where individuals are at high risk of attaining mosquito-borne diseases. Knowledge of mosquito species distributions and their viral infectivity are essential for assessing the risks of vector-borne infectious disease spread in the country. A previous systematic study of mosquito species composition revealed 81 mosquito species (22 genera and 42 subgenera) distributed in Vietnam [2]. Three genera (*Anopheles*, *Aedes*, and *Culex*) have been found to be potential vectors for mosquito-borne diseases locally. *Aedes* mosquitos are the main vector for DENV, CHIKV, and ZIKV. These are potentially severe fevers, and lethal clinical outcomes are possible. According to WHO recommendations, the key preventive measures for controlling mosquito-borne diseases are behavioral changes, increased public awareness, and environmental management [33].

In this study, the mosquito species composition in seven provinces of Vietnam was investigated based on sample collection over three phases in 2023. Species diversity was represented by the following mosquito genera: *Aedes* spp. (51.25%), *Culex* spp. (44.96%), *Anopheles* spp. (2.98%), and *Armigeres* spp. (0.81%). *Aedes* spp. predominated in Da Nang, Binh Dinh, Phu Yen, and Khanh Hoa provinces. *Culex* spp. predominated in Quang Nam, Quang Ngai, and Binh Thuan provinces [34,35].

The results obtained from this study provide evidence that *A. aegypti* is currently prevalent in all studied provinces. Previously published studies have cited *A. Aegypti* as one of the dominant species in mosquito populations [36,37,38]. *A. albopictus*, though less abundant, was still present, indicating its potential role as a secondary vector in certain areas. The distribution of mosquito species exhibited variation across provinces, likely influenced by urbanization, water storage practices, and local climatic conditions [7]. The obtained data on mosquito species diversity in specific Vietnamese provinces (southern and central regions) coincide with those obtained previously by other authors [2,7,39,40].

This study offers insights into the distribution, infection prevalence, and type composition of DENV in *Aedes* mosquito populations in central Vietnam. DENV viral RNA was detected in 92 of the collected pools. This represents 10.1% of all samples studied. The high level of dengue fever-carrying mosquito infestation in Vietnam may be a natural consequence of the country’s ecological and climatic conditions, coupled with socioeconomic challenges. The country experiences cyclical epidemics every 3–5 years, driven by factors such as viral evolution, collective immunity, and environmental changes [41].

The seasonal patterns observed in DENV infection prevalence align closely with the rainy season (May to November), when breeding conditions for mosquitoes are most favorable [42]. Provinces such as Quang Ngai, Phu Yen, and Khanh Hoa exhibited higher infection values, indicating that they are potential hotspots for dengue transmission. In contrast, areas like Binh Thuan and Da Nang showed no detectable DENV during certain study phases. This is likely due to effective local vector control measures or insufficient sample size.

This study identified three DENV types (DENV-1, DENV-2, and DENV-4) circulating in the survey area [43]. Provinces like Da Nang and Khanh Hoa, where multiple serotypes were detected, represent heightened risks for severe dengue fever cases due to ADE [44]. The ADE effect occurs when existing heterotypic antibodies bind, but fail to neutralize, virions of the subsequently infecting DENV type. This can potentially facilitate viral entry into host cells, thereby hindering the immune response [45,46]. While infection with a single serotype generally results in mild to moderate symptoms, the presence of multiple types within a geographical area has been shown to significantly increase the risk of severe dengue cases.

In addition to finding a high level of DENV prevalence in mosquitos of central Vietnam, ZIKV was detected in one sample from Quang Nam Province. CHIKV and ZIKV cases and episodes have indeed been reported in Vietnam. However, they occur at a significantly lower frequency compared to dengue fever and are typically reported as sporadic cases, or as part of serological investigations. Chikungunya was detected in several regions of Vietnam between 2017 and 2019, indicating that the virus is endemic. However, large-scale outbreaks have not been officially recorded [38]. As of 2017, only two cases of ZIKV infection among 8 105 children with fever were detected in central Vietnam, despite the active circulation of DENV [47]. Following the Zika epidemic, neutralizing antibodies were detected in a significant proportion of the population, indicating latent circulation of the virus according to a 2020 study [44]. Despite the high density of mosquito vectors, the number of ZIKV and CHIKV infections in Vietnam remains well below the number of dengue fever cases [38,48]. Larger studies are needed to accurately understand the mosquito infectivity of these viruses, and such studies are necessary to assess the risk of human infection with these viral fevers.

This study aligns with prior research in Vietnam and neighboring countries emphasizing the need for continuous surveillance of the spread of arboviruses in mosquito vectors [49].

The low detection frequency of CHIKV, ZIKV, and DENV-4 may reflect either a genuine feature of local arbovirus circulation or a methodological limitation due to sampling being restricted to three seasonal periods rather than year-round monitoring. In addition, the low detection rate of CHIKV and ZIKV in this study may be due to the low viral load, which did not reach the detection threshold of the reagents used. Due to logistical constraints, specimens were collected during the most representative climatic phases: the dry season, rainy season, and transitional period. Although full annual coverage was not feasible, this sampling design accounted for major seasonal weather variations, thereby minimizing potential biases in the epidemiological data.

## 5. Conclusions

This study provides information on mosquito species diversity in specific provinces of central Vietnam, as well as on the distribution of viruses among mosquitoes of the genus *Aedes*. The predominance of *A. aegypti* highlights its potential role as a primary vector for arboviruses in the region. The absence of certain genera in Da Nang may reflect ecological or anthropogenic influences on mosquito species distribution. The highest prevalence was observed for DENV. This indicates its dominant role in the structure of circulating arboviruses and suggests a significant risk of infection with this virus. Conducting routine, integrated surveillance (entomological and virological) makes it possible to track changes in mosquito vector abundance, species diversity, and their prevalence of infection with human pathogenic viruses. In summary, this study enhances our understanding of the ecological and epidemiological factors driving dengue transmission in central Vietnam. Continuous surveillance and adaptive strategies remain critical to managing the complexities of dengue epidemiology in the region.

## Data Availability

The original contributions presented in this study are included in the article/Supplementary Materials. Further inquiries can be directed to the corresponding author.

## Supplementary Materials

The following supporting information can be downloaded at https://www.mdpi.com/article/10.3390/v17070905/s1, Table S1: Mosquito collection data; Figure S1: Number of male and female mosquitos (*A. aegypti*, *A. albopictus*) in 2023; Figure S2: Heatmap of pools by province and phase.
