# Supplementary material for: Mosquito Species Diversity and Circulation of Mosquito-Borne Viruses in Selected Provinces of Central Vietnam
Source: Viruses. 2025 Jun 26;17(7):905. doi: 10.3390/v17070905 (PMC12299319; doi:10.3390/v17070905)
Supplement: Supplementary file 1 [file viruses-17-00905-s001.zip › Table S1. Mosquito Collection Data.pdf]

	Mosquito species	Number of mosquitoes					
		Phase 1		Phase 2		Phase 3	
		female	male	female	male	female	male
Da Nang	*A. aegypti*	77	79	117	186	98	38
	A. albopictus	x	x	2	x	x	x
	Anopheles spp.	x		x		x	
	C. quinquefasciatus	172		368		41	
	C. tritaeniorhynchus	x		x		x	
	Armigeres spp.	x		x		x	
Quang Nam	*A. aegypti*	189	310	56	76	100	73
	A. albopictus	1	1	6	1	x	x
	Anopheles spp.	x		64		4	
	C. quinquefasciatus	139		451		292	
	C. tritaeniorhynchus	x		x		x	
	Armigeres spp.	4		10		3	
Quang Ngai	*A. aegypti*	139	144	238	267	142	193
	A. albopictus	6	x	x	x	2	x
	Anopheles spp.	2		34		7	
	C. quinquefasciatus	545		322		344	
	C. tritaeniorhynchus	25		x		1	
	Armigeres spp.	15		16			
Binh Dinh	*A. aegypti*	170	203	175	267	139	204
	A. albopictus	10	20	10	20	5	x
	Anopheles spp.	20		69		14	
	C. quinquefasciatus	325		656		63	
	C. tritaeniorhynchus	x		x		x	
	Armigeres spp.	30		1		2	
Phu Yen	*A. aegypti*	141	167	226	202	143	241
	A. albopictus	2	x	x	x	x	x
	Anopheles spp.	27		122		x	
	C. quinquefasciatus	215		257		92	
	C. tritaeniorhynchus	5		x		x	
	Armigeres spp.	6		x		1	
Khanh Hoa	*A. aegypti*	96	54	124	74	172	199
	A. albopictus	21	x	x	x	x	x
	Anopheles spp.	x		5		3	
	C. quinquefasciatus	31		22		450	
	C. tritaeniorhynchus	x		x		x	
	Armigeres spp.	x		x		1	
Binh Thuan	*A. aegypti*	179	145	136	140	100	104
	A. albopictus	x	x	x	x	x	x
	Anopheles spp.	x		x		3	
	C. quinquefasciatus	456		151		198	
	C. tritaeniorhynchus	18		x		1	
	Armigeres spp.	13		x		x	
