# Supplementary figures and images for: Mosquito Species Diversity and Circulation of Mosquito-Borne Viruses in Selected Provinces of Central Vietnam
Source: Viruses. 2025 Jun 26;17(7):905. doi: 10.3390/v17070905 (PMC12299319; doi:10.3390/v17070905)

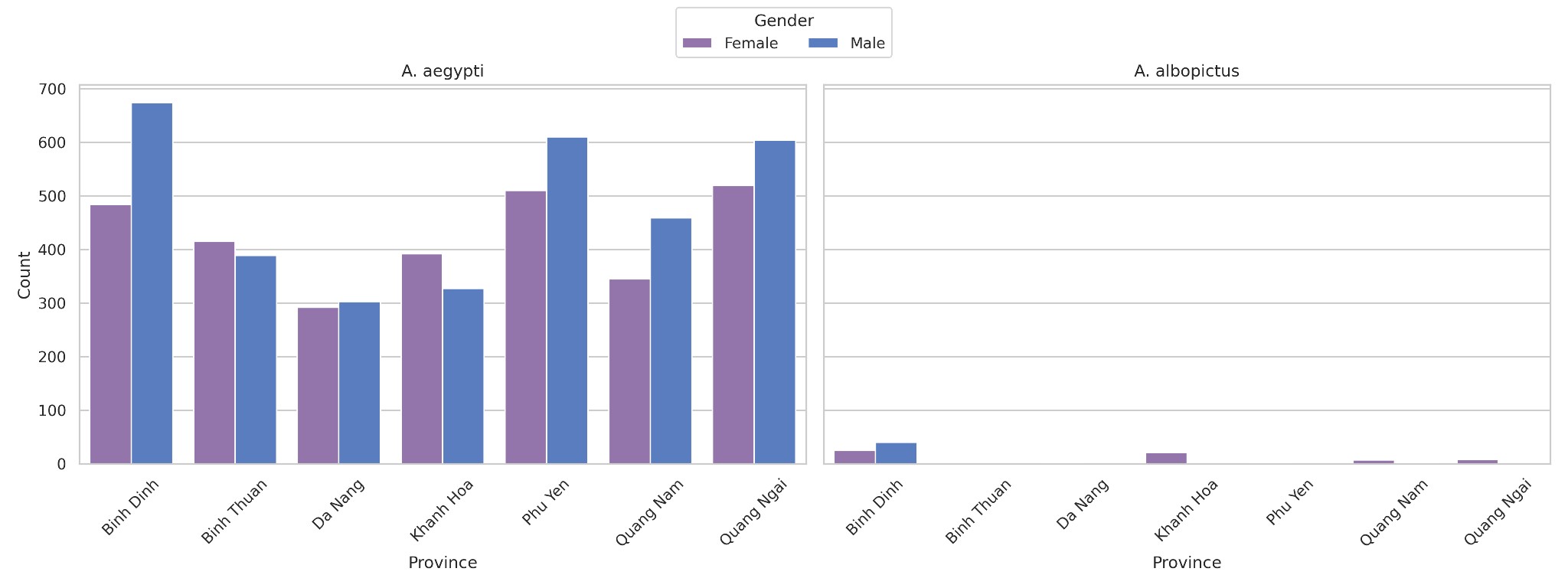

Supplement: Supplementary file 1 [file viruses-17-00905-s001.zip › Figure S2. Number of male and female mosquitos.jpg]

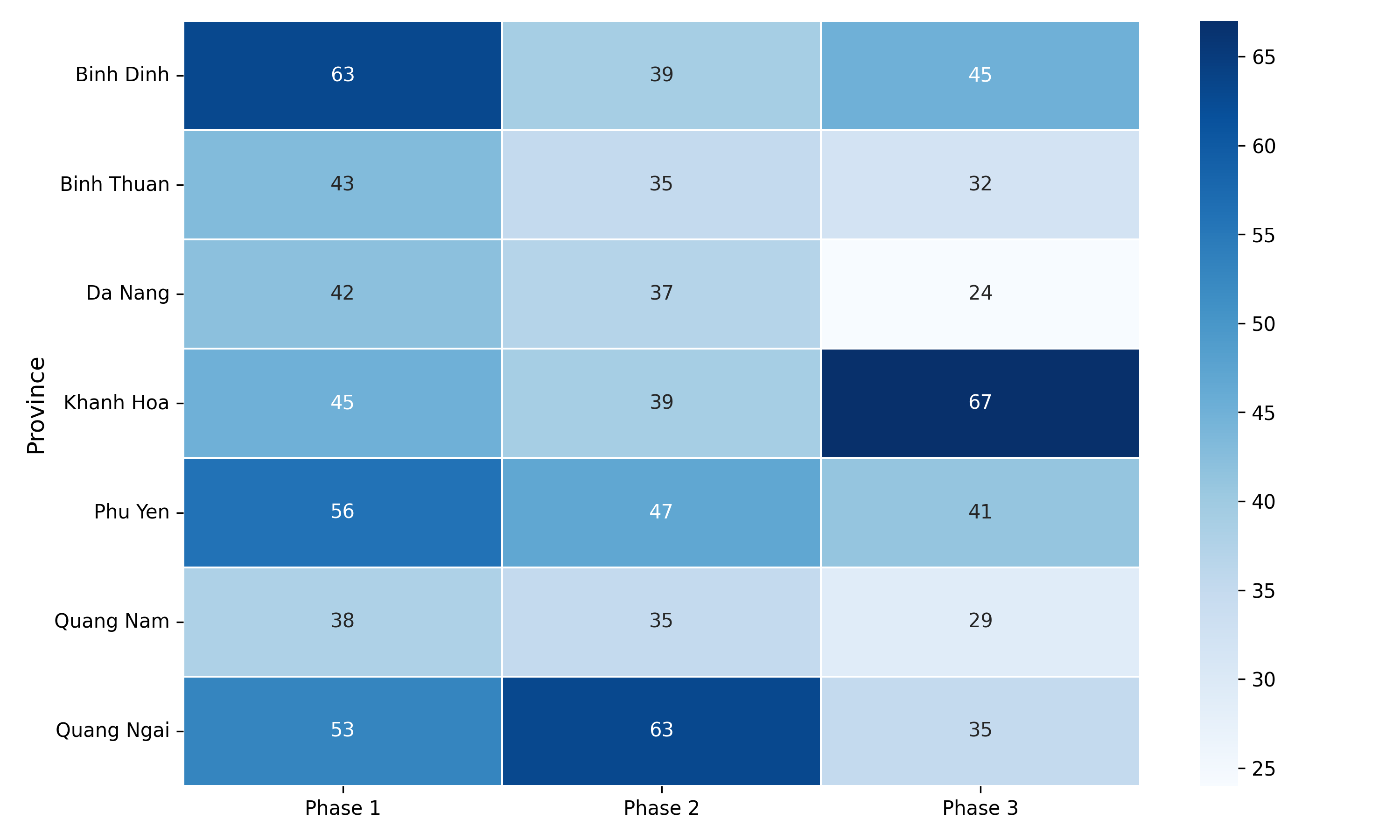

Supplement: Supplementary file 1 [file viruses-17-00905-s001.zip › Figure S3. Heatmap of pools by province and phase.png]
